# Bis(μ-chloroacetato-κ^2^ *O*:*O*′)bis(chloro­acetato-κ*O*)di-μ_3_-oxido-tetrakis[dibenzyl­tin(IV)]

**DOI:** 10.1107/S1600536810054000

**Published:** 2011-01-08

**Authors:** Jing Li, Handong Yin, Daqi Wang

**Affiliations:** a College of Chemistry and Chemical Engineering, Liaocheng University, Shandong 252059, People’s Republic of China

## Abstract

The title tetra­nuclear complex mol­ecule, [Sn_4_(C_7_H_7_)_8_(C_2_H_2_ClO_2_)_4_O_2_], has crystallographically imposed inversion symmetry. Each Sn atom has a distorted trigonal–bipyramidal geometry, with the equatorial plane formed by an oxido O atom and two C atoms of two benzyl anions. The configuration of the complex is stabilized by a pair of C—H⋯O hydrogen bonds. In the crystal, complex mol­ecules are linked into zigzag chains along [110] by C—H⋯O hydrogen bonds.

## Related literature

For the biological activity of organotin derivatives, see: Gielen *et al.* (1988 ▶). For a related structure, see: Teoh *et al.* (2002 ▶).
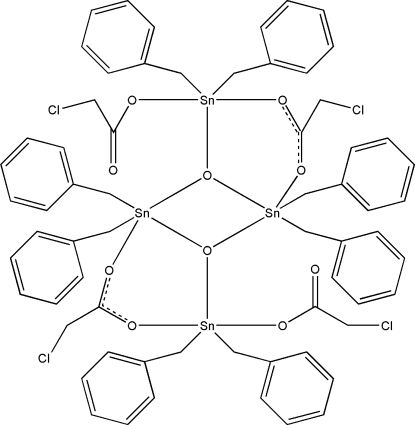


## Experimental

### 

#### Crystal data

[Sn_4_(C_7_H_7_)_8_(C_2_H_2_ClO_2_)_4_O_2_]
*M* *_r_* = 1609.71
Triclinic, 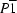

*a* = 10.4377 (8) Å
*b* = 13.0091 (9) Å
*c* = 13.3920 (11) Å
α = 104.920 (2)°
β = 103.208 (1)°
γ = 106.498 (1)°
*V* = 1593.0 (2) Å^3^
*Z* = 1
Mo *K*α radiation
μ = 1.77 mm^−1^
*T* = 298 K
0.20 × 0.13 × 0.08 mm

#### Data collection

Bruker SMART CCD diffractometer
Absorption correction: multi-scan (*SADABS*, Sheldrick, 1996 ▶) *T*_min_ = 0.718, *T*_max_ = 0.871
8386 measured reflections
5551 independent reflections
4110 reflections with *I* > 2σ(*I*)
*R*_int_ = 0.023

#### Refinement

*R*[*F* ^2^ > 2σ(*F* ^2^)] = 0.031
*wR*(*F* ^2^) = 0.054
*S* = 1.01
5551 reflections
370 parameters
H-atom parameters constrained
Δρ_max_ = 0.81 e Å^−3^
Δρ_min_ = −0.59 e Å^−3^

### 

Data collection: *SMART* (Siemens, 1996 ▶); cell refinement: *SAINT* (Siemens, 1996 ▶); data reduction: *SAINT*; program(s) used to solve structure: *SHELXS97* (Sheldrick, 2008 ▶); program(s) used to refine structure: *SHELXL97* (Sheldrick, 2008 ▶); molecular graphics: *SHELXTL* (Sheldrick, 2008 ▶); software used to prepare material for publication: *SHELXTL*.

## Supplementary Material

Crystal structure: contains datablocks I, global. DOI: 10.1107/S1600536810054000/rz2532sup1.cif

Structure factors: contains datablocks I. DOI: 10.1107/S1600536810054000/rz2532Isup2.hkl

Additional supplementary materials:  crystallographic information; 3D view; checkCIF report

## Figures and Tables

**Table 1:** Hydrogen-bond geometry (Å, °)

*D*—H⋯*A*	*D*—H	H⋯*A*	*D*⋯*A*	*D*—H⋯*A*
C11—H11⋯O4	0.93	2.49	3.229 (6)	136
C4—H4*A*⋯O4^i^	0.97	2.44	3.291 (5)	146

Symmetry code: (i) 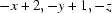.

**Fig. 1.**
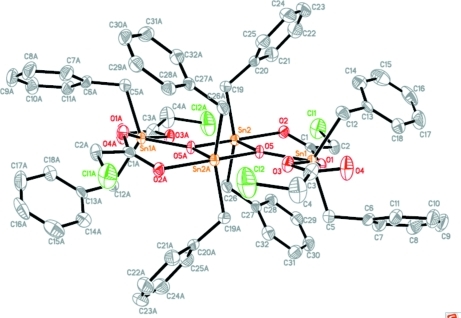
The molecular structure of the title compound, showing 50% probability displacement ellipsoids. H atoms have been omitted for clarity. Symmetry code: (A) 1-x, -y, -z.

**Fig. 2.**
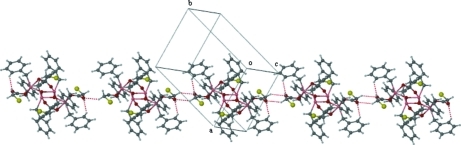
Crystal packing of the title compound, showing a one-dimensional chain formed by C—H···O hydrogen bonds (dashed lines).

## supplementary crystallographic information

### Comment

Organotin derivatives of carboxylic acid ligands have been extensively studied due to their biological activities (Gielen *et al.*, 1988). In our ongoing studies on chloroacetic acid and organotin, the title compound has been synthesized and we report herein its crystal structure.

The molecular structure of the tetranuclear title compound is shown in Fig. 1. Each Sn atom is five-coordinated by two carboxylic O atoms, an oxo O atom, and two C atoms of two benzyl anions into a distorted trigonal-bipyramidal geometry very similar to that observed in a related compound (Teoh *et al.*, 2002). The Sn—O distances are in the range 2.024 (2)–2.217 (2) Å. The conformation of the complex molecule is stabilized by a pair of C—H···O hydrogen bonds (Table 1). In the crystal (Fig. 2), complex molecules are connected along the [110] direction forming zigzag chains by C—H···O hydrogen bonds.

### Experimental

The reaction was carried out under nitrogen atmosphere. Chloroacetic acid (4 mmol) was added to a mixture of ethanol and benzene (1:3 *v*/*v*, 30 ml) with sodium ethoxide (4 mmol). The mixture was stirred for 0.5 h and then dichlorodibenzyltin (4 mmol) was added and the mixture was stirred at room temperature for 12 h. Crystals suitable for X-ray analysis were obtained by slow evaporation of a dichloromethane–petroleum ether (1:2 *v*/*v*) solution over a period of two weeks. Analysis, calculated for [(C_6_H_5_CH_2_)_8_(C_2_H_2_ClO_2_)_4_O_2_Sn_4_] (Mr = 1609.71): C 47.75, H 4.00%; found: C 47.82, H 3.95%.

### Refinement

All H atoms were positioned geometrically and refined as riding on their parent atoms, with C—H =  0.93–0.97 Å, and  with *U*_iso_(H) = 1.2 *U*_eq_(C).

### Crystal data

**Table:**

[Sn_4_(C_7_H_7_)_8_(C_2_H_2_ClO_2_)_4_O_2_]	*Z* = 1
*M**_r_* = 1609.71	*F*(000) = 796
Triclinic, *P*1	*D*_x_ = 1.678 Mg m^−^^3^
Hall symbol: -P 1	Mo *K*α radiation, λ = 0.71073 Å
*a* = 10.4377 (8) Å	Cell parameters from 3277 reflections
*b* = 13.0091 (9) Å	θ = 2.8–26.3°
*c* = 13.3920 (11) Å	µ = 1.77 mm^−^^1^
α = 104.920 (2)°	*T* = 298 K
β = 103.208 (1)°	Block, colourless
γ = 106.498 (1)°	0.20 × 0.13 × 0.08 mm
*V* = 1593.0 (2) Å^3^	

### Data collection

**Table:**

Bruker SMART CCD diffractometer	5551 independent reflections
Radiation source: fine-focus sealed tube	4110 reflections with *I* > 2σ(*I*)
graphite	*R*_int_ = 0.023
phi and ω scans	θ_max_ = 25.0°, θ_min_ = 1.7°
Absorption correction: multi-scan (*SADABS*, Sheldrick, 1996)	*h* = −12→12
*T*_min_ = 0.718, *T*_max_ = 0.871	*k* = −15→15
8386 measured reflections	*l* = −11→15

### Refinement

**Table:**

Refinement on *F*^2^	Primary atom site location: structure-invariant direct methods
Least-squares matrix: full	Secondary atom site location: difference Fourier map
*R*[*F*^2^ > 2σ(*F*^2^)] = 0.031	Hydrogen site location: inferred from neighbouring sites
*wR*(*F*^2^) = 0.054	H-atom parameters constrained
*S* = 1.01	*w* = 1/[σ^2^(*F*_o_^2^) + (0.0103*P*)^2^] where *P* = (*F*_o_^2^ + 2*F*_c_^2^)/3
5551 reflections	(Δ/σ)_max_ = 0.001
370 parameters	Δρ_max_ = 0.81 e Å^−^^3^
0 restraints	Δρ_min_ = −0.59 e Å^−^^3^

### Fractional atomic coordinates and isotropic or equivalent isotropic displacement parameters (Å^2^)

**Table:**

	*x*	*y*	*z*	*U*_iso_*/*U*_eq_	
Sn1	0.64849 (3)	0.28126 (2)	0.12506 (2)	0.03004 (9)	
Sn2	0.42902 (3)	−0.01234 (2)	0.09813 (2)	0.02829 (9)	
Cl1	0.36275 (18)	0.18206 (12)	0.44052 (12)	0.0831 (5)	
Cl2	0.8211 (2)	0.19683 (14)	−0.20870 (14)	0.1118 (7)	
O1	0.5054 (3)	0.2782 (2)	0.2235 (2)	0.0383 (8)	
O2	0.4600 (3)	0.1147 (2)	0.2565 (2)	0.0392 (8)	
O3	0.7429 (3)	0.2483 (2)	−0.0057 (2)	0.0379 (8)	
O4	0.8784 (4)	0.4294 (3)	0.0608 (3)	0.0715 (12)	
O5	0.5589 (3)	0.10884 (18)	0.0586 (2)	0.0285 (7)	
C1	0.4706 (4)	0.2167 (4)	0.2781 (3)	0.0315 (10)	
C2	0.4360 (5)	0.2750 (3)	0.3763 (3)	0.0454 (13)	
H2A	0.3700	0.3104	0.3532	0.055*	
H2B	0.5217	0.3351	0.4291	0.055*	
C3	0.8232 (5)	0.3343 (4)	−0.0136 (4)	0.0457 (13)	
C4	0.8540 (6)	0.3327 (4)	−0.1197 (4)	0.082 (2)	
H4A	0.9524	0.3793	−0.1021	0.098*	
H4B	0.7970	0.3673	−0.1571	0.098*	
C5	0.5445 (4)	0.3777 (3)	0.0550 (3)	0.0399 (12)	
H5A	0.5691	0.3829	−0.0096	0.048*	
H5B	0.4430	0.3385	0.0327	0.048*	
C6	0.5849 (5)	0.4954 (3)	0.1341 (4)	0.0417 (12)	
C7	0.5036 (6)	0.5190 (4)	0.1995 (4)	0.0570 (14)	
H7	0.4217	0.4615	0.1926	0.068*	
C8	0.5428 (7)	0.6268 (5)	0.2745 (5)	0.0718 (18)	
H8	0.4860	0.6415	0.3165	0.086*	
C9	0.6644 (7)	0.7124 (4)	0.2878 (5)	0.0757 (19)	
H9	0.6917	0.7842	0.3401	0.091*	
C10	0.7446 (6)	0.6917 (4)	0.2242 (4)	0.0681 (17)	
H10	0.8261	0.7501	0.2318	0.082*	
C11	0.7060 (5)	0.5837 (4)	0.1477 (4)	0.0536 (14)	
H11	0.7626	0.5706	0.1051	0.064*	
C12	0.8304 (4)	0.3392 (4)	0.2657 (3)	0.0464 (13)	
H12A	0.8584	0.2747	0.2687	0.056*	
H12B	0.9069	0.3945	0.2560	0.056*	
C13	0.8156 (4)	0.3925 (4)	0.3739 (4)	0.0396 (12)	
C14	0.8072 (5)	0.3359 (4)	0.4463 (4)	0.0579 (14)	
H14	0.8108	0.2630	0.4278	0.069*	
C15	0.7936 (6)	0.3835 (6)	0.5458 (5)	0.0804 (19)	
H15	0.7890	0.3431	0.5938	0.097*	
C16	0.7869 (6)	0.4895 (6)	0.5742 (5)	0.082 (2)	
H16	0.7772	0.5218	0.6413	0.098*	
C17	0.7944 (6)	0.5473 (5)	0.5041 (5)	0.080 (2)	
H17	0.7899	0.6198	0.5232	0.096*	
C18	0.8088 (5)	0.5004 (4)	0.4042 (4)	0.0581 (14)	
H18	0.8140	0.5416	0.3569	0.070*	
C19	0.5469 (4)	−0.0892 (3)	0.1865 (4)	0.0439 (12)	
H19A	0.4798	−0.1469	0.2018	0.053*	
H19B	0.5862	−0.1292	0.1374	0.053*	
C20	0.6649 (5)	−0.0187 (3)	0.2916 (4)	0.0375 (11)	
C21	0.6393 (6)	0.0059 (4)	0.3895 (4)	0.0545 (14)	
H21	0.5465	−0.0156	0.3897	0.065*	
C22	0.7473 (8)	0.0612 (5)	0.4867 (5)	0.0797 (19)	
H22	0.7268	0.0766	0.5517	0.096*	
C23	0.8841 (8)	0.0940 (5)	0.4894 (5)	0.087 (2)	
H23	0.9570	0.1303	0.5559	0.105*	
C24	0.9137 (6)	0.0732 (5)	0.3939 (6)	0.0766 (18)	
H24	1.0070	0.0963	0.3948	0.092*	
C25	0.8041 (5)	0.0174 (4)	0.2952 (4)	0.0532 (14)	
H25	0.8249	0.0041	0.2302	0.064*	
C26	0.2178 (4)	−0.0210 (3)	0.0304 (4)	0.0426 (12)	
H26A	0.1959	−0.0431	−0.0482	0.051*	
H26B	0.1548	−0.0822	0.0445	0.051*	
C27	0.1819 (4)	0.0827 (3)	0.0679 (4)	0.0348 (11)	
C28	0.1365 (4)	0.1037 (4)	0.1589 (4)	0.0433 (12)	
H28	0.1303	0.0535	0.1977	0.052*	
C29	0.1008 (5)	0.1986 (4)	0.1918 (4)	0.0530 (14)	
H29	0.0704	0.2117	0.2524	0.064*	
C30	0.1097 (5)	0.2725 (4)	0.1365 (4)	0.0578 (15)	
H30	0.0862	0.3363	0.1596	0.069*	
C31	0.1532 (5)	0.2540 (4)	0.0466 (4)	0.0554 (14)	
H31	0.1588	0.3047	0.0085	0.066*	
C32	0.1886 (4)	0.1591 (4)	0.0132 (4)	0.0473 (13)	
H32	0.2178	0.1466	−0.0480	0.057*	

### Atomic displacement parameters (Å^2^)

**Table:**

	*U*^11^	*U*^22^	*U*^33^	*U*^12^	*U*^13^	*U*^23^
Sn1	0.03285 (19)	0.02216 (15)	0.02845 (19)	0.00660 (14)	0.00873 (15)	0.00303 (14)
Sn2	0.03113 (18)	0.02562 (16)	0.02665 (19)	0.00911 (13)	0.01113 (15)	0.00652 (14)
Cl1	0.1243 (14)	0.0792 (10)	0.0594 (10)	0.0315 (10)	0.0595 (10)	0.0254 (9)
Cl2	0.1501 (17)	0.1002 (12)	0.0655 (12)	0.0180 (12)	0.0631 (12)	0.0037 (10)
O1	0.0420 (19)	0.0355 (16)	0.0381 (19)	0.0139 (14)	0.0196 (16)	0.0084 (15)
O2	0.059 (2)	0.0364 (17)	0.0247 (18)	0.0227 (15)	0.0155 (16)	0.0069 (15)
O3	0.0494 (19)	0.0318 (16)	0.0343 (19)	0.0119 (15)	0.0240 (16)	0.0085 (15)
O4	0.083 (3)	0.0391 (19)	0.072 (3)	−0.0024 (19)	0.038 (2)	0.0014 (19)
O5	0.0342 (16)	0.0197 (13)	0.0275 (16)	0.0057 (12)	0.0137 (13)	0.0026 (13)
C1	0.022 (2)	0.041 (3)	0.027 (3)	0.013 (2)	0.003 (2)	0.008 (2)
C2	0.053 (3)	0.045 (3)	0.033 (3)	0.017 (2)	0.020 (3)	0.002 (2)
C3	0.049 (3)	0.041 (3)	0.061 (4)	0.019 (2)	0.034 (3)	0.024 (3)
C4	0.105 (5)	0.062 (3)	0.072 (4)	0.001 (3)	0.054 (4)	0.024 (3)
C5	0.042 (3)	0.035 (2)	0.039 (3)	0.013 (2)	0.010 (2)	0.010 (2)
C6	0.056 (3)	0.033 (2)	0.039 (3)	0.022 (2)	0.012 (3)	0.013 (2)
C7	0.070 (4)	0.050 (3)	0.058 (4)	0.031 (3)	0.025 (3)	0.017 (3)
C8	0.109 (5)	0.064 (4)	0.064 (4)	0.054 (4)	0.041 (4)	0.018 (3)
C9	0.133 (6)	0.039 (3)	0.051 (4)	0.036 (4)	0.022 (4)	0.010 (3)
C10	0.091 (5)	0.038 (3)	0.060 (4)	0.010 (3)	0.018 (4)	0.015 (3)
C11	0.067 (4)	0.038 (3)	0.054 (4)	0.020 (3)	0.015 (3)	0.017 (3)
C12	0.037 (3)	0.056 (3)	0.041 (3)	0.017 (2)	0.011 (2)	0.009 (3)
C13	0.029 (3)	0.043 (3)	0.028 (3)	0.008 (2)	−0.005 (2)	−0.001 (2)
C14	0.063 (4)	0.059 (3)	0.049 (4)	0.024 (3)	0.013 (3)	0.017 (3)
C15	0.077 (5)	0.114 (5)	0.048 (4)	0.029 (4)	0.017 (4)	0.033 (4)
C16	0.060 (4)	0.108 (6)	0.037 (4)	0.015 (4)	0.007 (3)	−0.015 (4)
C17	0.079 (5)	0.062 (4)	0.071 (5)	0.027 (3)	0.014 (4)	−0.015 (4)
C18	0.065 (4)	0.051 (3)	0.045 (3)	0.019 (3)	0.008 (3)	0.007 (3)
C19	0.044 (3)	0.034 (2)	0.043 (3)	0.014 (2)	−0.001 (2)	0.011 (2)
C20	0.052 (3)	0.031 (2)	0.031 (3)	0.019 (2)	0.008 (2)	0.012 (2)
C21	0.070 (4)	0.048 (3)	0.048 (4)	0.018 (3)	0.020 (3)	0.022 (3)
C22	0.126 (6)	0.067 (4)	0.038 (4)	0.036 (4)	0.011 (4)	0.018 (3)
C23	0.108 (6)	0.059 (4)	0.056 (5)	0.023 (4)	−0.028 (4)	0.015 (4)
C24	0.052 (4)	0.069 (4)	0.088 (5)	0.016 (3)	−0.004 (4)	0.024 (4)
C25	0.059 (4)	0.050 (3)	0.055 (4)	0.023 (3)	0.020 (3)	0.020 (3)
C26	0.032 (3)	0.040 (3)	0.039 (3)	0.009 (2)	0.003 (2)	−0.001 (2)
C27	0.020 (2)	0.033 (2)	0.039 (3)	0.0040 (19)	0.004 (2)	0.004 (2)
C28	0.031 (3)	0.047 (3)	0.045 (3)	0.013 (2)	0.009 (2)	0.010 (2)
C29	0.042 (3)	0.063 (3)	0.046 (3)	0.025 (3)	0.013 (3)	0.000 (3)
C30	0.041 (3)	0.047 (3)	0.069 (4)	0.022 (3)	0.008 (3)	−0.003 (3)
C31	0.049 (3)	0.049 (3)	0.069 (4)	0.018 (3)	0.016 (3)	0.023 (3)
C32	0.040 (3)	0.051 (3)	0.046 (3)	0.015 (2)	0.017 (3)	0.010 (3)

### Geometric parameters (Å, °)

**Table:**

Sn1—O5	2.024 (2)	C13—C14	1.363 (6)
Sn1—C12	2.131 (4)	C13—C18	1.384 (6)
Sn1—C5	2.132 (4)	C14—C15	1.376 (6)
Sn1—O1	2.205 (3)	C14—H14	0.9300
Sn1—O3	2.206 (2)	C15—C16	1.359 (7)
Sn2—O5	2.037 (2)	C15—H15	0.9300
Sn2—C19	2.135 (4)	C16—C17	1.348 (8)
Sn2—C26	2.142 (4)	C16—H16	0.9300
Sn2—O5^i^	2.202 (2)	C17—C18	1.381 (6)
Sn2—O2	2.217 (2)	C17—H17	0.9300
Cl1—C2	1.759 (4)	C18—H18	0.9300
Cl2—C4	1.745 (5)	C19—C20	1.497 (5)
O1—C1	1.251 (4)	C19—H19A	0.9700
O2—C1	1.249 (4)	C19—H19B	0.9700
O3—C3	1.239 (4)	C20—C21	1.372 (6)
O4—C3	1.247 (5)	C20—C25	1.380 (6)
O5—Sn2^i^	2.202 (2)	C21—C22	1.367 (7)
C1—C2	1.514 (5)	C21—H21	0.9300
C2—H2A	0.9700	C22—C23	1.359 (8)
C2—H2B	0.9700	C22—H22	0.9300
C3—C4	1.523 (6)	C23—C24	1.361 (7)
C4—H4A	0.9700	C23—H23	0.9300
C4—H4B	0.9700	C24—C25	1.388 (7)
C5—C6	1.497 (5)	C24—H24	0.9300
C5—H5A	0.9700	C25—H25	0.9300
C5—H5B	0.9700	C26—C27	1.495 (5)
C6—C11	1.383 (6)	C26—H26A	0.9700
C6—C7	1.389 (6)	C26—H26B	0.9700
C7—C8	1.380 (6)	C27—C32	1.374 (6)
C7—H7	0.9300	C27—C28	1.397 (5)
C8—C9	1.369 (7)	C28—C29	1.384 (6)
C8—H8	0.9300	C28—H28	0.9300
C9—C10	1.354 (7)	C29—C30	1.352 (6)
C9—H9	0.9300	C29—H29	0.9300
C10—C11	1.391 (6)	C30—C31	1.369 (6)
C10—H10	0.9300	C30—H30	0.9300
C11—H11	0.9300	C31—C32	1.383 (6)
C12—C13	1.501 (5)	C31—H31	0.9300
C12—H12A	0.9700	C32—H32	0.9300
C12—H12B	0.9700		
			
O5—Sn1—C12	112.97 (14)	Sn1—C12—H12A	108.1
O5—Sn1—C5	117.57 (13)	C13—C12—H12B	108.1
C12—Sn1—C5	129.31 (16)	Sn1—C12—H12B	108.1
O5—Sn1—O1	88.99 (9)	H12A—C12—H12B	107.3
C12—Sn1—O1	92.54 (13)	C14—C13—C18	117.3 (4)
C5—Sn1—O1	84.79 (14)	C14—C13—C12	121.1 (4)
O5—Sn1—O3	79.27 (9)	C18—C13—C12	121.6 (5)
C12—Sn1—O3	100.37 (13)	C13—C14—C15	121.8 (5)
C5—Sn1—O3	92.56 (13)	C13—C14—H14	119.1
O1—Sn1—O3	165.15 (10)	C15—C14—H14	119.1
O5—Sn2—C19	110.68 (14)	C16—C15—C14	120.2 (6)
O5—Sn2—C26	107.18 (14)	C16—C15—H15	119.9
C19—Sn2—C26	142.10 (17)	C14—C15—H15	119.9
O5—Sn2—O5^i^	76.12 (9)	C17—C16—C15	119.2 (6)
C19—Sn2—O5^i^	92.46 (13)	C17—C16—H16	120.4
C26—Sn2—O5^i^	94.86 (13)	C15—C16—H16	120.4
O5—Sn2—O2	91.45 (9)	C16—C17—C18	121.1 (6)
C19—Sn2—O2	88.45 (13)	C16—C17—H17	119.5
C26—Sn2—O2	92.33 (13)	C18—C17—H17	119.5
O5^i^—Sn2—O2	167.03 (9)	C17—C18—C13	120.4 (5)
C1—O1—Sn1	131.6 (3)	C17—C18—H18	119.8
C1—O2—Sn2	129.1 (3)	C13—C18—H18	119.8
C3—O3—Sn1	115.4 (3)	C20—C19—Sn2	121.1 (3)
Sn1—O5—Sn2	133.37 (12)	C20—C19—H19A	107.1
Sn1—O5—Sn2^i^	122.44 (11)	Sn2—C19—H19A	107.1
Sn2—O5—Sn2^i^	103.88 (9)	C20—C19—H19B	107.1
O2—C1—O1	125.8 (4)	Sn2—C19—H19B	107.1
O2—C1—C2	119.9 (4)	H19A—C19—H19B	106.8
O1—C1—C2	114.3 (4)	C21—C20—C25	117.2 (5)
C1—C2—Cl1	113.8 (3)	C21—C20—C19	121.0 (5)
C1—C2—H2A	108.8	C25—C20—C19	121.6 (4)
Cl1—C2—H2A	108.8	C22—C21—C20	121.5 (5)
C1—C2—H2B	108.8	C22—C21—H21	119.2
Cl1—C2—H2B	108.8	C20—C21—H21	119.2
H2A—C2—H2B	107.7	C23—C22—C21	120.8 (6)
O3—C3—O4	123.6 (4)	C23—C22—H22	119.6
O3—C3—C4	121.5 (4)	C21—C22—H22	119.6
O4—C3—C4	114.8 (4)	C24—C23—C22	119.5 (6)
C3—C4—Cl2	114.5 (3)	C24—C23—H23	120.3
C3—C4—H4A	108.6	C22—C23—H23	120.3
Cl2—C4—H4A	108.6	C23—C24—C25	119.7 (6)
C3—C4—H4B	108.6	C23—C24—H24	120.1
Cl2—C4—H4B	108.6	C25—C24—H24	120.1
H4A—C4—H4B	107.6	C20—C25—C24	121.3 (5)
C6—C5—Sn1	111.6 (3)	C20—C25—H25	119.4
C6—C5—H5A	109.3	C24—C25—H25	119.4
Sn1—C5—H5A	109.3	C27—C26—Sn2	119.1 (3)
C6—C5—H5B	109.3	C27—C26—H26A	107.6
Sn1—C5—H5B	109.3	Sn2—C26—H26A	107.6
H5A—C5—H5B	108.0	C27—C26—H26B	107.6
C11—C6—C7	117.4 (4)	Sn2—C26—H26B	107.6
C11—C6—C5	121.8 (4)	H26A—C26—H26B	107.0
C7—C6—C5	120.8 (4)	C32—C27—C28	117.5 (4)
C8—C7—C6	120.8 (5)	C32—C27—C26	121.7 (4)
C8—C7—H7	119.6	C28—C27—C26	120.8 (4)
C6—C7—H7	119.6	C29—C28—C27	120.4 (5)
C9—C8—C7	120.7 (5)	C29—C28—H28	119.8
C9—C8—H8	119.6	C27—C28—H28	119.8
C7—C8—H8	119.6	C30—C29—C28	120.5 (5)
C10—C9—C8	119.5 (5)	C30—C29—H29	119.8
C10—C9—H9	120.3	C28—C29—H29	119.8
C8—C9—H9	120.3	C29—C30—C31	120.5 (5)
C9—C10—C11	120.5 (5)	C29—C30—H30	119.7
C9—C10—H10	119.8	C31—C30—H30	119.7
C11—C10—H10	119.8	C30—C31—C32	119.3 (5)
C6—C11—C10	121.1 (5)	C30—C31—H31	120.4
C6—C11—H11	119.5	C32—C31—H31	120.4
C10—C11—H11	119.5	C27—C32—C31	121.8 (4)
C13—C12—Sn1	116.7 (3)	C27—C32—H32	119.1
C13—C12—H12A	108.1	C31—C32—H32	119.1
			
O5—Sn1—O1—C1	45.3 (3)	C7—C8—C9—C10	2.0 (9)
C12—Sn1—O1—C1	−67.7 (4)	C8—C9—C10—C11	−1.5 (9)
C5—Sn1—O1—C1	163.1 (4)	C7—C6—C11—C10	0.2 (7)
O3—Sn1—O1—C1	82.8 (6)	C5—C6—C11—C10	−177.8 (5)
O5—Sn2—O2—C1	38.0 (3)	C9—C10—C11—C6	0.5 (8)
C19—Sn2—O2—C1	148.6 (4)	O5—Sn1—C12—C13	−108.9 (3)
C26—Sn2—O2—C1	−69.3 (4)	C5—Sn1—C12—C13	66.4 (4)
O5^i^—Sn2—O2—C1	54.4 (7)	O1—Sn1—C12—C13	−18.9 (3)
O5—Sn1—O3—C3	178.8 (3)	O3—Sn1—C12—C13	168.5 (3)
C12—Sn1—O3—C3	−69.5 (4)	Sn1—C12—C13—C14	106.5 (4)
C5—Sn1—O3—C3	61.2 (3)	Sn1—C12—C13—C18	−73.0 (5)
O1—Sn1—O3—C3	140.5 (4)	C18—C13—C14—C15	−0.5 (7)
C12—Sn1—O5—Sn2	79.0 (2)	C12—C13—C14—C15	−180.0 (5)
C5—Sn1—O5—Sn2	−96.9 (2)	C13—C14—C15—C16	0.6 (9)
O1—Sn1—O5—Sn2	−13.3 (2)	C14—C15—C16—C17	−0.3 (10)
O3—Sn1—O5—Sn2	175.8 (2)	C15—C16—C17—C18	0.0 (10)
C12—Sn1—O5—Sn2^i^	−108.46 (17)	C16—C17—C18—C13	0.1 (9)
C5—Sn1—O5—Sn2^i^	75.62 (19)	C14—C13—C18—C17	0.1 (7)
O1—Sn1—O5—Sn2^i^	159.21 (15)	C12—C13—C18—C17	179.6 (4)
O3—Sn1—O5—Sn2^i^	−11.65 (14)	O5—Sn2—C19—C20	58.2 (4)
C19—Sn2—O5—Sn1	−99.1 (2)	C26—Sn2—C19—C20	−124.6 (3)
C26—Sn2—O5—Sn1	82.6 (2)	O5^i^—Sn2—C19—C20	134.3 (4)
O5^i^—Sn2—O5—Sn1	173.5 (3)	O2—Sn2—C19—C20	−32.8 (4)
O2—Sn2—O5—Sn1	−10.2 (2)	Sn2—C19—C20—C21	86.8 (5)
C19—Sn2—O5—Sn2^i^	87.36 (15)	Sn2—C19—C20—C25	−97.7 (5)
C26—Sn2—O5—Sn2^i^	−90.86 (14)	C25—C20—C21—C22	−1.7 (7)
O5^i^—Sn2—O5—Sn2^i^	0.0	C19—C20—C21—C22	174.0 (4)
O2—Sn2—O5—Sn2^i^	176.25 (12)	C20—C21—C22—C23	0.1 (9)
Sn2—O2—C1—O1	−21.5 (6)	C21—C22—C23—C24	1.2 (9)
Sn2—O2—C1—C2	156.7 (3)	C22—C23—C24—C25	−1.0 (9)
Sn1—O1—C1—O2	−32.2 (6)	C21—C20—C25—C24	1.9 (7)
Sn1—O1—C1—C2	149.5 (3)	C19—C20—C25—C24	−173.7 (4)
O2—C1—C2—Cl1	−7.2 (5)	C23—C24—C25—C20	−0.7 (8)
O1—C1—C2—Cl1	171.2 (3)	O5—Sn2—C26—C27	−61.4 (4)
Sn1—O3—C3—O4	18.4 (6)	C19—Sn2—C26—C27	121.3 (4)
Sn1—O3—C3—C4	−157.4 (4)	O5^i^—Sn2—C26—C27	−138.4 (3)
O3—C3—C4—Cl2	−21.1 (7)	O2—Sn2—C26—C27	30.8 (4)
O4—C3—C4—Cl2	162.7 (4)	Sn2—C26—C27—C32	93.4 (5)
O5—Sn1—C5—C6	157.5 (3)	Sn2—C26—C27—C28	−88.4 (4)
C12—Sn1—C5—C6	−17.6 (4)	C32—C27—C28—C29	−0.3 (6)
O1—Sn1—C5—C6	71.4 (3)	C26—C27—C28—C29	−178.7 (4)
O3—Sn1—C5—C6	−123.3 (3)	C27—C28—C29—C30	−0.2 (7)
Sn1—C5—C6—C11	84.2 (5)	C28—C29—C30—C31	0.5 (8)
Sn1—C5—C6—C7	−93.7 (5)	C29—C30—C31—C32	−0.3 (7)
C11—C6—C7—C8	0.3 (8)	C28—C27—C32—C31	0.5 (6)
C5—C6—C7—C8	178.2 (5)	C26—C27—C32—C31	178.9 (4)
C6—C7—C8—C9	−1.3 (9)	C30—C31—C32—C27	−0.2 (7)

Symmetry codes: (i) −*x*+1, −*y*, −*z*.

### Hydrogen-bond geometry (Å, °)

**Table:**

*D*—H···*A*	*D*—H	H···*A*	*D*···*A*	*D*—H···*A*
C11—H11···O4	0.93	2.49	3.229 (6)	136
C4—H4A···O4^ii^	0.97	2.44	3.291 (5)	146

Symmetry codes: (ii) −*x*+2, −*y*+1, −*z*.
